# Expression and interactions of stereochemically active lone pairs and their relation to structural distortions and thermal conductivity

**DOI:** 10.1107/S2052252520003619

**Published:** 2020-03-31

**Authors:** Kasper Tolborg, Carlo Gatti, Bo B. Iversen

**Affiliations:** a Center for Materials Crystallography, Department of Chemistry and iNANO, Aarhus University, Langelandsgade 140, Aarhus 8000, Denmark; b CNR-SCITEC Istituto di Scienze e Tecnologie Chimiche ‘Giulio Natta’, via Golgi Section, via Golgi 19, Milano 20133, Italy

**Keywords:** lone pairs, chemical bonding, thermal conductivity, quantum crystallography, electron densities

## Abstract

In chemistry, stereochemically active lone pairs are typically described as an important non-bonding effect, and recent interest has centred on understanding the derived effect of lone pair expression on physical properties such as thermal conductivity. To manipulate such properties, it is essential to understand the conditions that lead to lone pair expression and provide a quantitative chemical description of their identity to allow comparison between systems. Here, density functional theory calculations are used first to establish the presence of stereochemically active lone pairs on antimony in the archetypical chalcogenide MnSb_2_O_4_. The lone pairs are formed through a similar mechanism to those in binary post-transition metal compounds in an oxidation state of two less than their main group number [*e.g.* Pb(II) and Sb(III)], where the degree of orbital interaction (covalency) determines the expression of the lone pair. In MnSb_2_O_4_ the Sb lone pairs interact through a void space in the crystal structure, and their their mutual repulsion is minimized by introducing a deflection angle. This angle increases significantly with decreasing Sb—Sb distance introduced by simulating high pressure, thus showing the highly destabilizing nature of the lone pair interactions. Analysis of the chemical bonding in MnSb_2_O_4_ shows that it is dominated by polar covalent interactions with significant contributions both from charge accumulation in the bonding regions and from charge transfer. A database search of related ternary chalcogenide structures shows that, for structures with a lone pair (Sb*X*_3_ units), the degree of lone pair expression is largely determined by whether the antimony–chalcogen units are connected or not, suggesting a cooperative effect. Isolated Sb*X*_3_ units have larger *X*—Sb—*X* bond angles and therefore weaker lone pair expression than connected units. Since increased lone pair expression is equivalent to an increased orbital interaction (covalent bonding), which typically leads to increased heat conduction, this can explain the previously established correlation between larger bond angles and lower thermal conductivity. Thus, it appears that for these chalcogenides, lone pair expression and thermal conductivity may be related through the degree of covalency of the system.

## Abstract 2

Stereochemically active lone pairs are an important non-bonding effect with significant implications on crystal structure and physical properties. This work reveals the highly repulsive nature of interactions between stereochemically active lone pairs, and shows how cooperative effects lead to stronger expression of stereochemically active lone pairs, affecting their impact on thermal conductivity.

## 1. Introduction   

Stereochemically active lone pairs are usually treated as textbook examples of non-bonding effects and occur in post-transition metal compounds in which the post-transition metal is in an oxidation state of two lower than its main group number, such as Pb(II) and Sb(III). This means that the two outermost *s* electrons are available to form a lone pair, leading to, after hybridization, the possibility of an asymmetric coordination environment. These oxidation states are found in many technologically important materials, such as thermoelectric materials (Snyder & Toberer, 2008 ▸; Zhao *et al.*, 2014 ▸), multiferroics (Ramesh & Spaldin, 2007 ▸), phase-change materials (Lencer *et al.*, 2008 ▸) and optoelectronics (Ogo *et al.*, 2008 ▸; Zhou *et al.*, 2015 ▸). But their structural chemistry is quite intriguing, since the ability to form a lone pair does not always lead to asymmetric coordination, *e.g.* in symmetric rock salt PbS and asymmetric litharge PbO (Walsh & Watson, 2005*b* ▸). Originally, and still in most textbooks, stereochemically active lone pairs are described as on-site *sp* hybridization on the metal atom, which is the origin of its name as a ‘chemically inactive’ (*i.e.* non-bonding) but ‘stereochemically active’ (*i.e.* structure-determining) effect. However, this does not fully explain the anion dependence on the tendency to form an asymmetric coordination environment.

In a series of articles, Walsh, Watson and co-workers introduced a revised model to account for this anion dependence based also on work by Waghmare and co-workers (Walsh *et al.*, 2011 ▸; Watson *et al.*, 1999 ▸; Watson & Parker, 1999 ▸; Walsh & Watson, 2005*a* ▸,*b* ▸; Waghmare *et al.*, 2003 ▸). They showed that the lone pairs are in fact not chemically inactive, but rather that the *s* orbital on the metal atom mixes with the anion *p* orbital to form a bonding and an anti-bonding state, followed by mixing of the anti-bonding state with the metal *p* states. The tendency to form a lone pair is therefore highly dependent on the energy difference between the metal valence states and the anion valence *p* states. If this difference is large, the formation of the bonding orbital is less favourable, and thus the lone pair is not ‘expressed’ and the higher symmetry structure is adopted (Walsh *et al.*, 2011 ▸). Thus, the expression of a lone pair is highly dependent on covalent interactions between cation and anion.

With these trends established, we have a framework for understanding the structures of materials with the possibility of forming stereochemically active lone pairs. The relation to physical properties has been investigated for thermoelectric materials, where the presence of oxidation states, which are able to form stereochemically active lone pairs, was shown to lead to a decrease in lattice thermal conductivity in the Cu–Sb–Se ternary system, *i.e.* Sb^3+^ systems have lower thermal conductivity than Sb^5+^ systems (Skoug & Morelli, 2011 ▸). Furthermore, an empirical correlation between bond angle and lattice thermal conductivity in As, Sb and Bi chalcogenides in oxidation state +3 was established with larger bond angles generally leading to lower thermal conductivity. The ability to form a lone pair has also been used to describe the good electronic properties for thermoelectricity of rock salt lead and tin monochalcogenides compared with other rock salt materials (Zeier *et al.*, 2016 ▸). Interestingly, it is often not the formation of a stereochemically active lone pair, but merely the presence of the investigated oxidation states that result in favourable thermoelectric properties; in some cases, the expression of a lone pair is even detrimental for either electronic or thermal properties in closely related systems (Cagnoni *et al.*, 2018 ▸; Yu *et al.*, 2020 ▸).

However, the general relation to physical properties and the crystal structures beyond the asymmetric local coordination environment is still poorly understood. The structural point is illustrated by the large difference in crystal structures adopted by otherwise similar materials, where the local coordination environment is asymmetric, such as the localized molecular units in Sb_2_O_3_ and the infinite chains in Sb_2_S_3_. Similarly, the bond angles around the stereochemically active lone pair can be very different for otherwise similar systems (Wang & Liebau, 1996 ▸; Skoug & Morelli, 2011 ▸).

**Figure 1:**
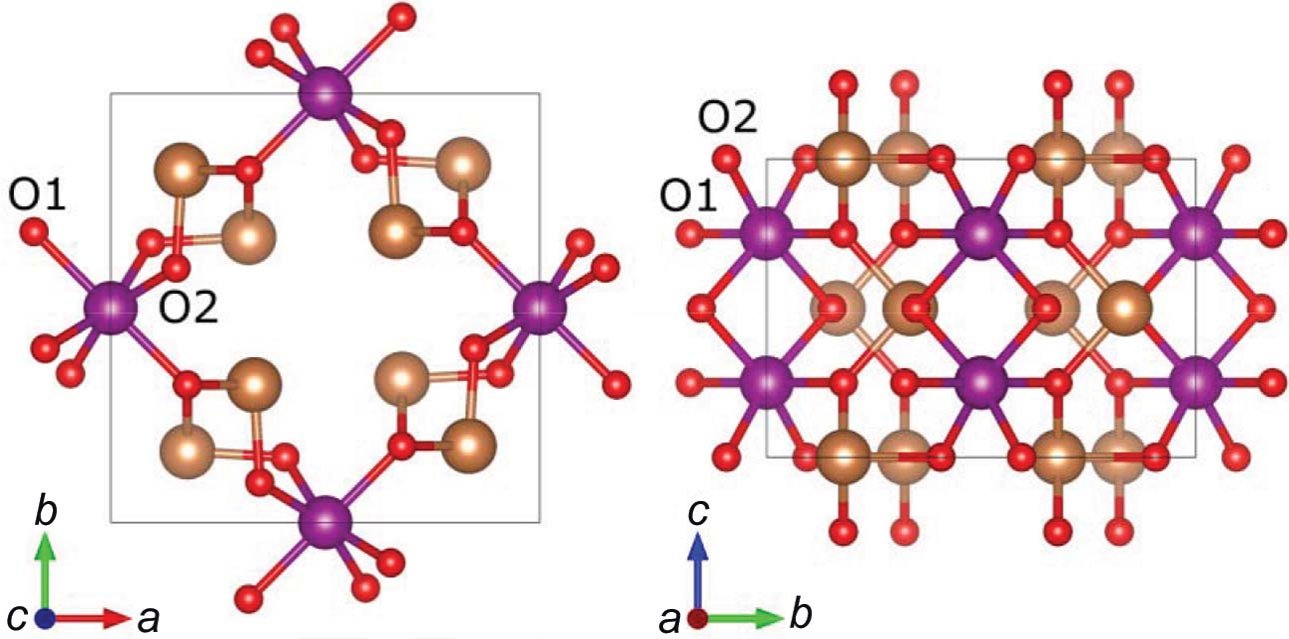
Crystal structure of MnSb_2_O_4_ viewed along the *c* axis (left) and *a* axis (right). Mn is shown as purple, Sb as brown and O as red. The two crystallographically unique oxygen atoms are marked as O1 and O2.

An interesting chalcogenide material in which the local coordination environment is asymmetric is the isostructural group of ternary oxides *M*Sb_2_O_4_, where *M* is a transition metal. Structures have been reported for *M* = Mn, Fe, Co, Ni and Zn, crystallizing in the space group *P*4_2_/*mbc*, isostructural to red lead, Pb_3_O_4_, which can formally be written as Pb(IV)Pb(II)_2_O_4_ to highlight the similarity between the groups. The crystal structure of MnSb_2_O_4_ is shown in Fig. 1 ▸ along the *c* and *a* axes. The structure consists of distorted MnO_6_ octahedra with two long Mn—O1 and four short Mn—O2 distances, and SbO_3_ units in a trigonal pyramidal coordination with two long Sb—O1 and one short Sb—O2 distances, leaving room for a presumed lone pair on antimony to occupy the fourth corner in a tetrahedron (Müller-Buschbaum, 2003 ▸; Roelsgaard *et al.*, 2016 ▸).

The *M*Sb_2_O_4_ compounds are relatively unexplored in the literature, but they have been studied for potential use in Li-ion batteries due to presence of channels of low electron density along the *c* axis, and magnetic structures have been studied and were shown to result in different orderings depending on the metal atom (Fjellvåg *et al.*, 1985 ▸; Jibin *et al.*, 2012 ▸; Roelsgaard *et al.*, 2016 ▸; Nørby *et al.*, 2016 ▸). Our present interest in this class of materials arises from the interesting structure, where the presumed position of the antimony lone pair based on the coordination environment suggests that two lone pairs point almost directly towards each other. Basic chemical intuition would suggest that this interaction should be highly unfavourable, and similar structural motifs are not, to our knowledge, adopted by other materials with stereochemically active lone pairs.

Here we investigate the expression and interactions between the presumed stereochemically active lone pairs on antimony in MnSb_2_O_4_ through density functional theory (DFT) calculations based on both orbital and electron density related descriptors to gain further understanding of this intriguing structural motif. Furthermore, we simulate the high-pressure behaviour of the material to force the lone pairs closer together and investigate the response of this external stimulus. Then, we report a characterization of all other bonding interactions in the material, and finally, we perform a database analysis on a group of related structures in order to derive general features regarding the influence of stereochemically active lone pairs on crystal structures, and their relation to physical properties, particularly thermal conductivity.

## 2. Computational details   

Periodic *ab initio* DFT calculations on MnSb_2_O_4_ were performed in *CRYSTAL14* using the POB-TZVP basis set (Dovesi *et al.*, 2014 ▸; Laun *et al.*, 2018 ▸; Peintinger *et al.*, 2013 ▸). This basis set uses a full-potential all-electron basis for Mn and O and a small-core effective core potential for Sb with 23 electrons in the valence corresponding to the 4*s* ^2^4*p* ^6^4*d* ^10^5*s* ^2^5*p* ^3^ electrons. The PBE0 hybrid functional was used and reciprocal space was sampled on an 8 × 8 × 8 grid in a Monkhorst–Pack net in the first Brillouin zone (Adamo & Barone, 1999 ▸; Perdew *et al.*, 1996 ▸). The initial geometry and magnetic symmetry were set to the geometry from Roelsgaard *et al.* (2016 ▸) and the antiferromagnetic configuration corresponding to the A-mode from Fjellvåg *et al.* (1985 ▸). In principle, this results in lowering the space group symmetry to 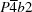, which would require displacement of all atomic *z* coordinates by 1/4 to follow the standard settings of the space groups. However, in this case it was achieved using the *CRYSTAL14* keyword MODISYMM to remove (half of) the symmetry elements to maintain the coordinates corresponding to those in the structural space group. A ferromagnetic configuration was also tried, but resulted in higher energy and similar bonding features.

First, the full geometry, *i.e.* cell and atomic coordinates, was optimized with the symmetry from the magnetic structure. The resulting atomic coordinates still followed the structural space group symmetry within the numerical error. Results of the optimization are given in the supporting information. Here it is seen that the deviation of the optimized geometry from the experimental structure at 100 K is less than 0.5% for cell parameters and less than 2% for bond lengths. After this, a series of unit-cell volumes from 10% smaller to 4% larger than the equilibrium volume in steps of 2% were constructed and the cell parameters and atomic coordinates were relaxed at constant volume (CVOLOPT keyword). The energy–volume curve was fitted to a third-order Birch–Murnaghan equation-of-state to find the corresponding pressures.

The chemical bonding was analysed in terms of projected density of states and valence electron density as implemented in *CRYSTAL14*, and topological analysis following Bader’s Quantum Theory of Atoms in Molecules (QTAIM) (Bader, 1990 ▸) using *TOPOND* interfaced with *CRYSTAL14* (Gatti *et al.*, 1994 ▸).

## 3. Results and discussion   

### 3.1. Density of states and valence electron density   

**Figure 2:**
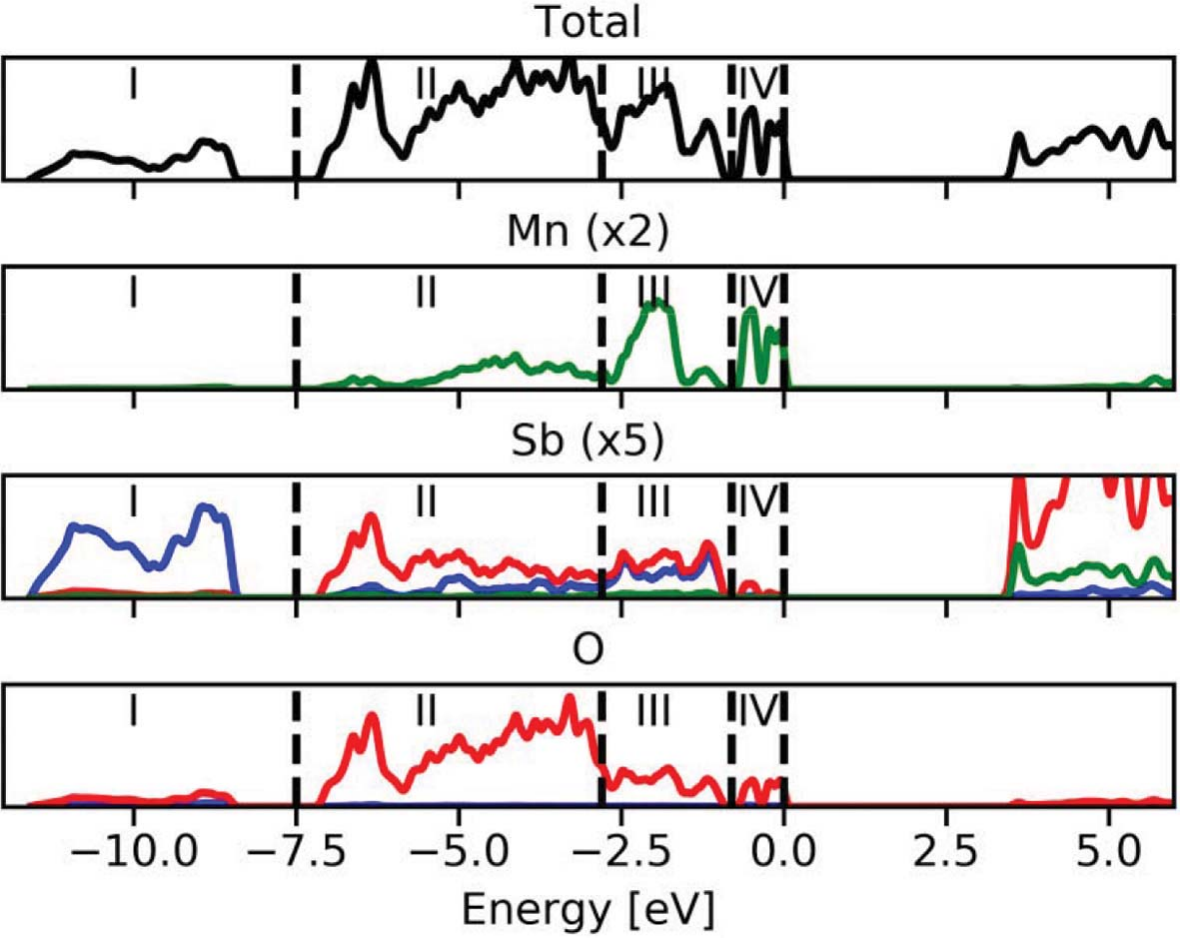
Total and orbital projected electronic density of states. Blue lines are *s* states, red lines are *p* states and green lines are *d* states. Individual atomic contributions have been enlarged to allow visual inspection.

To establish the presence of a stereochemically active lone pair on antimony, we first investigate the orbital projected density of states (DOS) and the valence electron density in the region of interest. The DOS (Fig. 2 ▸) can be divided into four distinct regions in accordance with previously established stereochemically active lone pairs in Sb_2_O_3_ and tin monochalcogenides (Allen *et al.*, 2013 ▸; Walsh & Watson, 2005*a* ▸). Region IV is special for this material compared with, for example, Sb_2_O_3_ due to the transition metal and consists mainly of manganese *d* states and oxygen *p* states. A similar feature is also seen in region III, but here we also observe a large degree of overlap between oxygen *p* states and antimony *s* and *p* states. Further below the Fermi level, we have region II, which consists mainly of oxygen *p* states and antimony *p* states, and region I, which consists mainly of antimony *s* states and oxygen *p* states. Regions I, II and III are qualitatively very similar to the ones in, for example, Sb_2_O_3_, with the complication of the presence of a transition metal here (Allen *et al.*, 2013 ▸).

**Figure 3:**
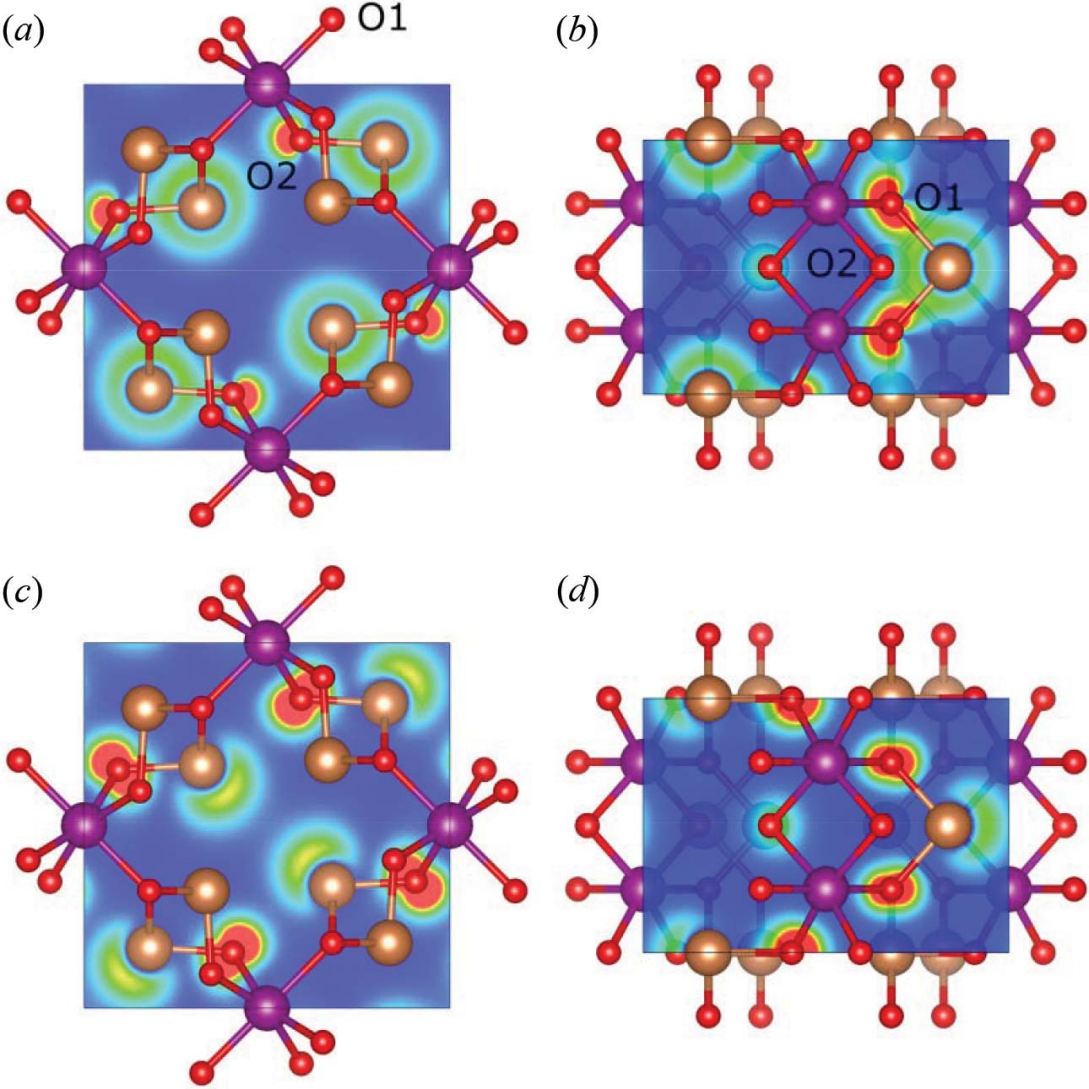
Valence electron density in selected planes and regions from Fig. 2 ▸. (*a*) and (*b*) Region I, (*c*) and (*d*) Region III. (*a*) and (*c*) (001) plane at *z* = 1/2, (*b*) and (*d*) plane spanned by Sb and two equivalent O1 atoms. Contours are drawn from 0 (blue) to 0.0445 e bohr^−3^ (red). Mn is shown as purple, Sb as brown and O as red, and the two crystallographically unique O atoms are again marked as O1 and O2 in (*a*) and (*b*). The figures were generated using *VESTA* (Momma & Izumi, 2008 ▸).

To further highlight the character of these regions, we can plot the valence electron density within these energy intervals. The most interesting regions regarding the stereochemically active lone pair are regions I and III, which are shown in Fig. 3 ▸. In Figs. 3 ▸(*a*) and 3 ▸(*b*), we see that region I consists of a bonding interaction between Sb and O, both to O1 and to O2. However, we clearly observe that the electron density in this energy range is higher for the long Sb—O1 bond. This oxygen is bonded to two Sb and one Mn, which seems to affect the energy levels of its valence states. This indicates that the interaction between Sb and O1 is the dominating feature for the expression of the lone pair.

In region III [Figs. 3 ▸(*c*) and 3 ▸(*d*)] the lone pair on Sb and localized electron density on O are clearly observed. This region is identified as the key for the stereochemically active lone pair, and this valence density is in very good correspondence with the density observed in, for example, SnO by Walsh & Watson (2005*a* ▸), which is one of the archetypical examples of stereochemically active lone pairs induced by the anion. Thus, it is safe to conclude that the present material has stereochemically active lone pairs on antimony that follow the established framework, although with some complexity induced by the presence of the transition metal causing, for example, the different oxygen atoms to behave quite differently.

### 3.2. Real space identification of the lone pairs   

So far, we have considered the electronic structure from an orbital projected point of view. However, it is interesting also to analyse the lone pairs and the chemical bonding from the perspective of real space descriptors, assessing, for example, the electron localization. A lone pair is characterized by a large degree of electron localization, and it is thus commonly identified using descriptors that assess the localization of electrons or the concentration of the electron density. The two commonly used descriptors are the electron localization function (ELF) (Becke & Edgecombe, 1990 ▸; Silvi & Savin, 1994 ▸; Savin *et al.*, 1997 ▸) and the Laplacian of the electron density, ∇^2^ρ(*r*) (Gatti, 2005 ▸), respectively.

**Figure 4:**
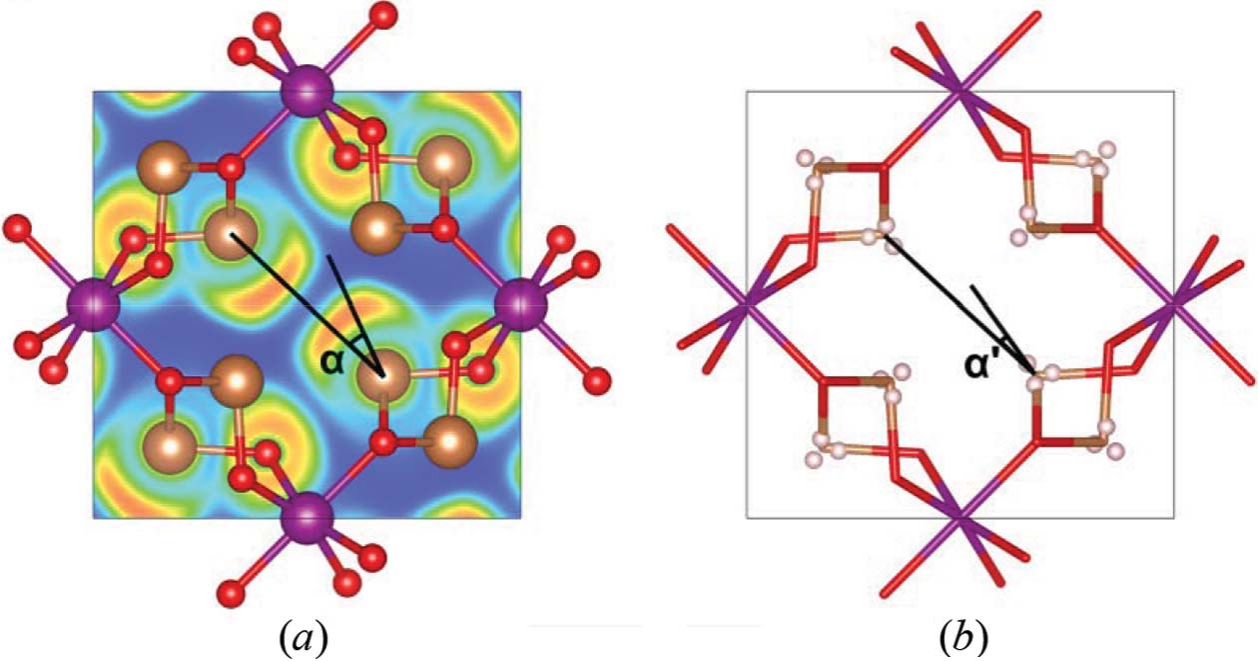
(*a*) ELF in the (001) plane at *z* = 1/2. Contours are drawn from 0 (blue) to 1 (red). (*b*) Maxima in *L*(*r*) = −∇^2^ρ(*r*) in the N-shell of Sb. The deviation of the lone pair maxima from the interatomic line in the (*a*) ELF and (*b*) *L*(*r*) are indicated by the angles α and α′, respectively. Mn is shown as purple, Sb as brown and O as red.

In Fig. 4 ▸(*a*), the ELF is plotted in the (001) plane at *z* = 1/2, *i.e.* at the plane containing the two antimony atoms pointing towards each other. The electrons appear to be extremely localized at the expected lone pair region on antimony; furthermore, we see a tendency of the lone pairs to avoid each other as the maximum localizations are located at a significant angle away from the interatomic line, *i.e.* angle α in Fig. 4 ▸(*a*). It is also interesting to note that not only do the ELF maxima tend to avoid each other, but the lone pairs are themselves asymmetric. This leads to alignment of high and low electron localizations, which minimizes the unfavourable interactions between the lone pairs.

In Fig. 4 ▸(*b*), the lone pair is visualized using the Laplacian of the electron density. If plotting it in a 2D plane like the ELF, no obvious features are seen, so instead, we search for critical points in the charge concentration on antimony. Typically, one would perform the search in the outermost valence shell, but for heavy atoms, this is often buried within the charge depletions of the inner shells (Shi & Boyd, 1988 ▸). Therefore, we use the procedure outlined by Sist *et al.* (2017 ▸), who showed that the lone pair character is not only present as a charge concentration in the outermost valence shell, but also in the shell below. In Fig. 4 ▸(*b*), the four charge concentrations in the *N*-shell on Sb are shown, and they correspond well to an *sp* ^3^ hybridization with three concentrations pointing towards neighbouring oxygen atoms, and one charge concentration pointing in the direction where the lone pairs were also observed from the ELF. Also, from the charge concentrations, a slight deviation from the interatomic line is observed as indicated by the angle α′.

### 3.3. Pressure effects on lone pair interaction   

**Figure 5:**
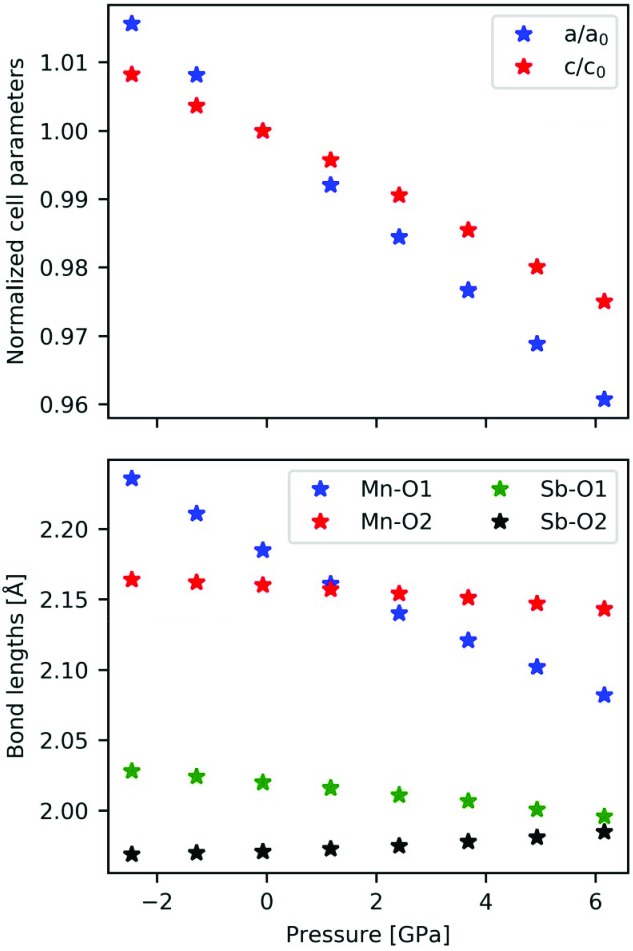
Normalized cell parameters and bond lengths of the four unique short bonds as a function of pressure. The optimized geometry used in the previous section is seen to be at slightly low negative pressure from a Birch–Murnaghan third-order fit to the energy–volume curve.

We have now shown that there is a destabilizing interaction due to lone pairs pointing towards each other, which is presumably decreased by introducing an angle between the lone pairs. To further investigate this feature, we simulated the effect of pressure on the structure by optimizing the structure at various constant volumes and extracting the pressure from the energy–volume relation. In Fig. 5 ▸, the unit-cell parameters and bond lengths are shown as a function of pressure. We can see that the *a* axis contracts more with increased pressure, which makes sense since the channels of low electron density run along the *c* axis, meaning that there is a void space for the structure to relax into in the *ab* plane. Interestingly, we see that the bond lengths change significantly with pressure, where the originally short Mn—O2 bond remains almost constant with pressure, whereas the Mn—O1 bond length decreases significantly with pressure. Eventually, the order of the bond lengths switches at a very moderate pressure between 1 and 2 GPa. This is a consequence of the Mn—O1 bond lying in the *ab* plane, which contracts the most. Here it should be noted that the difference between the Mn—O1 and Mn—O2 bond lengths at zero pressure is smaller in our optimization than from experiment at 100 K, so experimentally the switch may not occur until at a higher pressure (see Table S1 of the supporting information). For the Sb—O bonds, the changes are less significant, but interestingly enough the short Sb—O2 bond, which lies in the *ab* plane, actually increases slightly as a function of pressure, whereas the longer Sb—O1 bond decreases slightly. It is assumed that no phase transition occurs, although a phase transition has been observed for the iron analogue, FeSb_2_O_4_, at around 4 GPa (Hinrichsen *et al.*, 2006 ▸).

**Figure 6:**
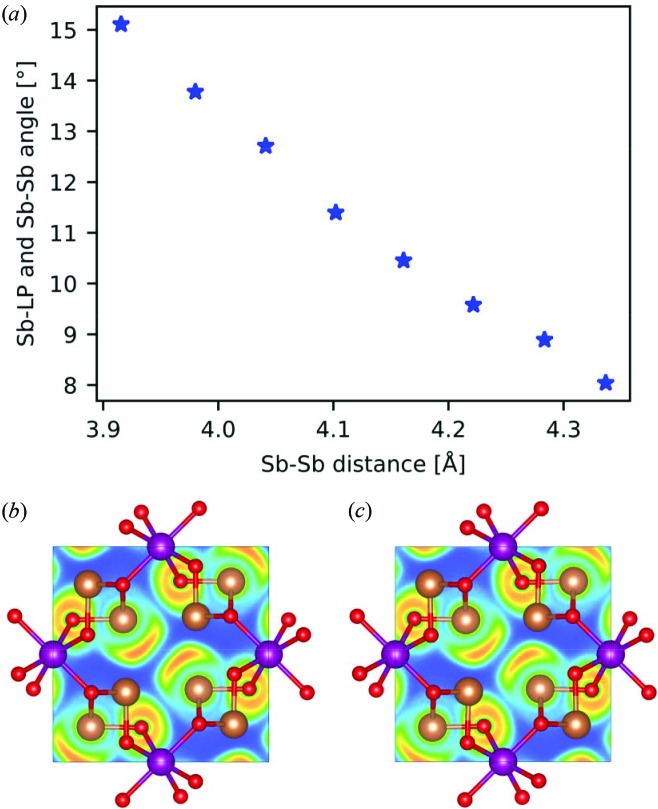
(*a*) Angle between the Sb to lone pair vector and the Sb to Sb vector as a function of pressure. The angle is indicated as α′ in Fig. 4 ▸(*b*) for the equilibrium volume. (*b*) and (*c*) ELF in the (001) plane at *z* = 1/2 for the structure with a volume reduction of (*b*) 6% and (*c*) 10%. Contours are drawn from 0 (blue) to 1 (red); (*b*) and (*c*) are compared with Fig. 4 ▸(*a*) at equilibrium volume.

In Fig. 6 ▸(*a*), the effect of pressure on the lone pair deflection gauged by the maxima in *L*(*r*) = −∇^2^ρ(*r*) is shown [angle α′ in Fig. 4 ▸(*b*)]. It is clear that, with decreasing distance between Sb atoms resulting from the high pressure, the angle between the interatomic vector and the lone pair vector increases. The enhanced angle with increasing pressure (and a shorter Sb—Sb distance) is also qualitatively visualized in Figs. 6 ▸(*b*) and 6 ▸(*c*), where the ELF is seen at a volume reduced by 6 and 10%, respectively, to enable comparison with the ELF for the optimized volume shown in Fig. 4 ▸(*a*). This deflection is a clear effect of the repulsion between lone pairs, which use the flexibility of the structure to reduce the repulsion as much as possible.

### 3.4. Topological analysis of the electron density   

Having analysed the interactions between the lone pairs on antimony, we now report the general bonding scheme in the material. Since the electron density distribution is a quantum mechanical observable, it is useful to analyse the chemical bonding (almost) purely based on this, which is the foundation of Bader’s QTAIM (Bader, 1990 ▸). Here we define the topological atom based on partitioning of the electron density using the zero-flux surface, and we define a bonding interaction based on the (3, −1) critical points, *i.e.* points where the density is at the minimum along one direction (parallel to the bond) and the maximum along the other two directions (perpendicular to the bond). Based on the properties at the bond critical point (BCP), we can obtain valuable information about chemical bonding based on well established relations that can often be related to structural and physical properties (Gatti, 2005 ▸; Tolborg & Iversen, 2019 ▸).

In Table 1 ▸, all BCPs are shown. BCPs are found at all four of the unique bonds drawn in Fig. 1 ▸, *i.e.* two unique Mn—O and two unique Sb—O bonds. In addition, BCPs are found between Sb and O2 at a larger distance and between the two Sb atoms ‘through’ the lone pair. All bonds have quite low, but non-negligible density and a positive Laplacian, which is, however, not surprising since the BCPs lie in a region of charge depletion on the heavy atoms and at a distance from the nuclei where the charge distribution of the corresponding isolated atoms is also depleted (Shi & Boyd, 1988 ▸). Looking at the energy densities, we see that all four short bonds have negative total energy densities, and generally the shorter the bond, the larger the electron density and the more negative the total energy density. Furthermore, the kinetic energy per electron, *G*(*r*_b_)/ρ(*r*_b_), is slightly above one. All these characteristics are common for polar covalent bonds and donor–acceptor bonds (Gatti, 2005 ▸). The integrated atomic charges in Table 2 ▸ show that large ionic contributions are present in all interactions, since charges are found to be +1.53 and +1.89 on Mn and Sb, respectively. These charges are quite high, showing the large degree of ionic bonding, although significantly smaller than those corresponding to their formal oxidation states.

**Figure 7:**
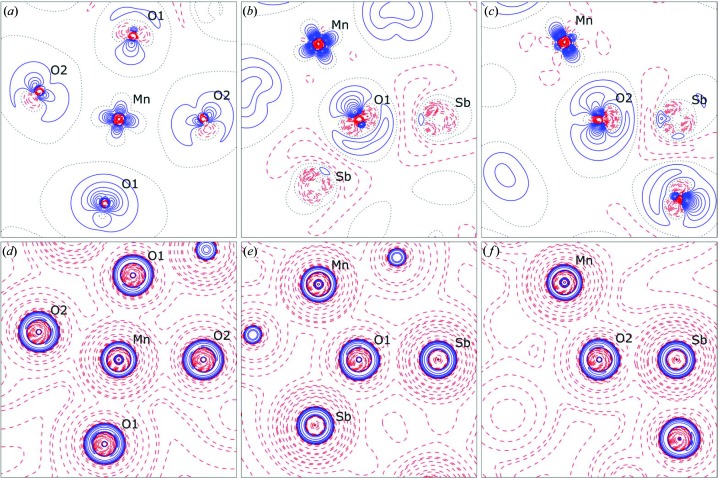
(*a*)–(*c*) Deformation densities in the planes containing the labelled nuclei; positive (solid blue), negative (dashed red) and zero (dotted black) contours are shown and contour spacings are 0.1 e Å^−3^ in (*a*) and 0.05 e Å^−3^ in (*b*) and (*c*). (*d*)–(*f*) Negative Laplacian of the electron density in selected planes; positive (solid blue), negative (dashed red) and zero (dotted black) contours are shown at *a* × 10^*n*^, where *a* = 1, 2, 4, 8 and *n* = −2, −1, 0, 1, 2, 3, 4.

In Fig. 7 ▸, the deformation density and negative Laplacian of the electron density are shown for selected planes. Here, we observe that there is some charge accumulation in all four types of short bonds. It is especially interesting to note that manganese shows positive deformation density towards oxygen, which clearly highlights the covalent contribution to this bond. In the Sb—O bonds, the deformation density is less pronounced and both positive and negative deformation density are observed along the bond path. This is in very good agreement with the observation from both theory and experiment in Sb_2_O_3_ (Whitten *et al.*, 2004 ▸). Combined with the fact that charge concentrations on antimony point towards oxygen, this shows that there is some degree of covalency in this bonding, meaning that the Sb—O bonds are of polar covalent type.

Another reasonable interpretation of the chemical bonding would be in terms of charge-shift bonding (Shaik *et al.*, 2009 ▸; Shaik *et al.*, 2020 ▸). Here the important contribution to the bond energy is the resonance energy between ionic and covalent valence bond (electronic) structures, and this is generally found to lead to a small, but non-negligible electron density and a positive Laplacian at the BCP. For light main-group elements, the distinction between covalent and charge-shift bonding is easily made, since a negative Laplacian is expected for a (polar) covalent bond. In the present case, since a negative Laplacian is not expected for a (polar) covalent bond involving heavy elements, we cannot unequivocally assign these bonds to either polar covalent or charge-shift bonds without resorting to a valence bond model wavefunction analysis. In either case, there is a significant degree of covalency and therefore orbital overlap in the chemical bonding, which is important for reconciling the orbital-based view with our analysis of the electron density, since the theory of stereochemically active lone pairs requires significant orbital overlap.

The two extra BCPs found are between atoms at a much longer distance, the density is much lower and the total energy density is practically zero. This shows that they are largely present due to the geometry dictated by the stronger interactions, rather than being important structure-determining features on their own. The Sb—Sb BCP in particular is clearly not a stabilizing interaction, since we have shown that the charge concentrations try to avoid each other to lower the repulsion between adjacent lone pairs, rather than directing them towards each other as seen in typical covalent bonds.

### 3.5. Implications for other materials with stereochemically active lone pairs   

The clear repulsion between stereochemically active lone pairs, which we have demonstrated here, might have important implications for our understanding of crystal structures with expressed stereochemically active lone pairs. In almost all other structures, an important structural motif is that lone pairs point into areas where no other lone pairs are present such as in the litharge structure adopted by SnO and PbO, and the GeS structure adopted by GeS, GeSe, SnS and SnSe. Even in a structure like cubic Sb_2_O_3_ (senarmonite), where the lone pair region points towards other Sb atoms, they arrange in a tetrahedron to avoid having charge concentrations pointing directly towards each other (Walsh *et al.*, 2011 ▸). In the present structure, the lone pairs are forced into the same region in pairs, but tend to decrease this unfavourable interaction by introducing a deflection angle. Furthermore, the lone pairs only interact strongly between pairs of atoms, but each of these atomic pairs tend to occupy mutually exclusive regions of space to decrease interactions with other atomic pairs, similarly to the individual behaviour in the other structures mentioned. Thus, the repulsive nature of the lone pairs has an important structure determining role, which often leads to the formation of structural motifs such as layers, channels or cages.

As mentioned earlier, there are indications that the unique oxygen atom bonded to two antimony atoms was much more involved in the lone pair formation than the other, which is only bonded to one Sb atom. This suggests that there could be a cooperative effect involved in the lone pair expression. Skoug & Morelli (2011 ▸) showed an empirical correlation between bond angles and lattice thermal conductivity in chalcogenides of As, Sb and Bi. In this case, bond angles were used as a measure for the degree of lone pair expression according to valence shell electron pair repulsion (VSEPR) theory, since a more strongly expressed lone pair closer to the nuclei will give rise to smaller bond angles. In their example case of the Cu–Sb–Se system, the main structural difference between the two materials CuSbSe_2_ and Cu_3_SbSe_3_, where the lone pair is more strongly expressed in the former, is that the SbSe_3_ units are connected in CuSbSe_2_, but isolated in Cu_3_SbSe_3_. These two observations motivated us to perform a database search in the Inorganic Crystal Structure Database (ICSD) (Bergerhoff *et al.*, 1983 ▸) in order to understand whether it is a general trend that isolated units tend to have larger bond angles arising from weaker lone pair expression, and therefore possibly lower lattice thermal conductivity as a result. The search included all *M*Sb(III)*X* structures (where *M* is an alkali metal, an alkaline-earth metal or a transition metal, and *X* is O, S or Se) from the ICSD, where the coordination number of antimony is three. Structures with partial occupancies (*i.e.* disorder) and structures where the atomic coordinates were not refined were excluded. The structures were sorted based on whether the Sb*X*_3_ units were isolated or connected. In order to make this distinction, an operational definition of a bond, which depends only on the geometry, must be used. We tried a criterion based on covalent radii, but to include a reasonable number of bonds an arbitrary increase of the sum of covalent radii must be used. Instead we chose to use a criterion based on bond valence parameters, *s* = exp[(*r*_0_ − *r*)/*B*], where *B* = 0.37 is a universal constant, *r*_0_ is the tabulated bond valence parameter, *r* is the bond length and *s* is the corresponding bond valence (Brown & Altermatt, 1985 ▸; Brese & Keeffe, 1991 ▸). It was suggested by Altermatt & Brown (1985 ▸) that values larger than 0.6 for the bond valence correspond to a covalent bond, and values larger than 0.038× the oxidation state of the cation correspond to a bond to be included in the calculation of the effective valence. However, these two numbers are too strict and too loose, respectively, in the present case for a reasonable number of bonds that should be included, so we chose a bond valence value of 0.3 to be the minimum value for a bond to be included in the analysis. This corresponds to maximum distances of 2.42, 2.90 and 3.02 Å for bonds between Sb and O, S and Se, respectively. Changing the parameters slightly makes a small difference in the final histogram, but does not affect the conclusions.

**Figure 8:**
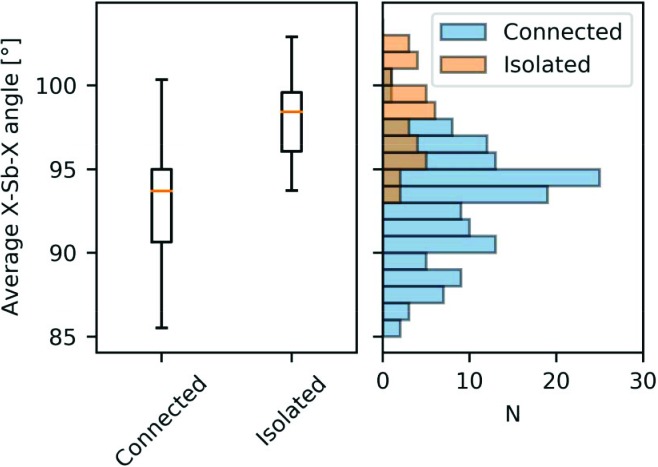
Average *X*—Sb—*X* angle for *M*Sb(III)*X* structures (where *M* is an alkali metal, an alkaline earth metal or a transition metal, and *X* is O, S or Se) from the ICSD, depending on whether the Sb*X*_3_ units are connected or isolated. To the left a box plot is shown with an orange bar marking the median value, the box marks the interquartile range and the whiskers mark the spread of the data points. To the right a histogram shows the number of Sb*X*_3_ units (*N*) with bond angles within the given interval. Data are binned in 1° intervals. The search is based on 77 crystal structures with 137 unique connected Sb*X*_3_ units and 35 unique isolated Sb*X*_3_ units. See Table S2 for a list of structures and ICSD codes.

Fig. 8 ▸ shows that, despite a significant degree of overlap between the two groups, there is a clear tendency for isolated Sb*X*_3_ units to have larger bond angles than the connected ones. According to VSEPR theory, this means that the lone pair is further from the nuclei in the isolated cases, or put differently, the lone pair is less expressed. Therefore, the present results suggest that there is a cooperative effect involved in the stronger expression of the lone pair, meaning the ability of the chalcogen to be involved in lone pair expression becomes stronger when it is influenced by more than one Sb atom. Since the bond angles, and thus lone pair expression, are strongly correlated with lattice thermal conductivity, this gives an interesting handle for designing new low thermal conductivity structures with potential applications as thermoelectric materials.

As discussed above, lone pair expression arises from an orbital interaction, *i.e.* a covalent chemical interaction, so the larger degree of lone pair expression should correspond to a more covalent interaction. It is well known that stronger bonding leads to larger phonon velocities and therefore larger lattice thermal conductivity (Slack, 1973 ▸). This also means that heat conduction mainly occurs along covalent bonds, which was quantified for a layered system by Zhang *et al.* (2018 ▸), who found a strong correlation between the electron density at the bond critical points and the thermal conductivity in presumably layered structures. Thus, we would expect that weaker orbital interaction should lead to lowering of the thermal conductivity. Therefore, an explanation for the lower lattice thermal conductivity in systems with larger bond angles shown by Skoug & Morelli (2011 ▸) may arise from weaker covalent interactions in these systems, originating from whether the units are connected or isolated, and indeed, they showed that Cu_3_SbSe_3_ with isolated Sb*X*_3_ units has lower thermal conductivity than CuSbSe_2_ with connected units.

Previously, the differences in bond angles have been interpreted as differences in effective atomic valence of the Sb atom (Wang & Liebau, 1996 ▸). Here a perfect tetrahedron with four neighbours (bond angles of 109.5°) corresponds to an oxidation state of +5 and a complete transfer of the 5*s* lone pair to a bonding interaction with an anion, whereas an oxidation state of +3 in a threefold coordination corresponds to a completely localized lone pair and thus a small bond angle. Intermediate cases then correspond to a progressive change in effective oxidation state and a less localized lone pair, which results in a larger bond angle. This was based on the fact that shorter bond lengths, and thus formally a larger effective valence, were observed for larger bond angles. Alternatively, one can imagine the limit of a flat coordination with 120° angles corresponding to an *sp* ^2^-hybridized Sb with a lone pair in the remaining *p* orbital. Progressive changes towards small bond angles correspond to more *s* character in the lone pair, and correspondingly more *p* character in the Sb—*X* bonds, which leads to longer bonds, similar to the increase in the C—H bond lengths with increasing *p* character in going from acetylene (*sp*) through ethylene (*sp* ^2^) to ethane (*sp* ^3^) (Bent, 1961 ▸), and in agreement with previous observations (Wang & Liebau, 1996 ▸). Interestingly, it is the smaller bond angles corresponding to the longer bonds that give rise to the largest thermal conductivity. Also in the case of MnSb_2_O_4_, it is in fact the longer Sb—O bond that has the largest degree of covalency determined from the valence electron densities and is more involved in lone pair formation. The present group of materials is thus a counterexample to the otherwise established correlation between shorter bond lengths and higher thermal conductivity, which is present over a very wide range of bond lengths and thermal conductivities, but with several groups of similar systems not following the trend (Zeier *et al.*, 2016 ▸). Thus, it seems to be the covalency of an interaction, rather than the bond length itself, which is important for the thermal conductivity.

The lattice thermal conductivity of a material is determined from several contributions, which in simple terms can be divided into two groups: those arising from the phonon dispersion itself (*e.g.* phonon group velocity) and those arising from the scattering of phonons (*e.g.* phonon–phonon scattering from anharmonicity or impurity scattering). Lone pair expression is often discussed in terms of the presence of asymmetric coordination, and the repulsion between the lone pair and valence electrons on neighbouring atoms, which is thought to lead to an anharmonic vibration potential, and therefore a decreased thermal conductivity through phonon–phonon scattering (Skoug & Morelli, 2011 ▸; Zhang *et al.*, 2012 ▸). With the present observations of increased covalency for connected units and the correspondence between lone pair expression and covalency, we instead suggest that lone pair expression and thermal conductivity are mainly related through the covalency of the system, and not necessarily because of specific anharmonic potential directions. This means that for weakly expressed lone pairs, the smaller degree of covalency should give rise to lower phonon velocities and a less rigid electronic structure, leading to lower thermal conductivity.

An interesting limiting case for our discussion is structures with a coordination number of six in perfect octahedral symmetry such as the rock salt lead chalcogenides, where the lone pair is not expressed in the static structure. During atomic displacement, the lone pair will be weakly expressed in different spatial regions depending on the direction of vibration, corresponding to a large coupling between atomic displacement and electronic structure. This leads to dynamic local distortions in this type of materials (Bozin *et al.*, 2010 ▸; Sangiorgio *et al.*, 2018 ▸). The bonding in these materials has been classified as resonant bonding (Lencer *et al.*, 2008 ▸; Shportko *et al.*, 2008 ▸), and this has been suggested as the origin of their low thermal conductivity by leading to phonon softening and strong anharmonicity (Lee *et al.*, 2014 ▸). More recently, the concept was termed metavalent bonding, and it was shown that displacement of GeTe along its Peierls distortion is associated with more covalent bonding and a more rigid electronic structure showed by a decrease in response properties such as the Born effective charge (Raty *et al.*, 2019 ▸). This distortion can be seen as a transition starting in the ideal symmetric rock-salt structure, where simple electron counting and the electron sharing calculated by Raty *et al.* (2019 ▸) reveal the formation of six 2-centre-1-electron (2c-1e) bonds between Ge and Te formed by their *p* orbitals, while the remaining valence electrons are localized on the atoms in the spherically symmetric *s* orbitals. Upon displacement of Ge along the [111] direction, three short and three long bonds are formed. In the completely distorted limit, the bonding situation approaches three 2c-2e bonds. In this case, due to the loss of symmetry, the last two electrons on Ge will no longer be distributed spherically and symmetrically, but rather form an expressed lone pair as the last corner in a tetrahedron, resembling the bonding situation in GeSe (Sist *et al.*, 2017 ▸). Thus, the consequence of approaching covalent bonding from metavalent bonding in a system like GeTe is also expression of a stereochemically active lone pair.

In this way, the relation between bond angle and thermal conductivity found by Skoug & Morelli (2011 ▸) can be considered as a progressive change from covalent bonding (strong lone pair expression, small bond angle) towards metavalent or resonant bonding (weak lone pair expression, large bond angle), which leads to lowering of the thermal conductivity. Interestingly, as we have shown here, the tendency to form small or large bond angles is strongly influenced by whether the Sb*X*_3_ units are connected or isolated.

The strong coupling between atomic displacement and electronic structure may give rise to anharmonicity, for example, in thermoelectric SnSe where a specific anharmonic mode revealed from inelastic neutron scattering was shown to highly perturb the electronic structure in the regions of interest for the lone pair (Li *et al.*, 2015 ▸). Similarly, anharmonicity has also been revealed both experimentally and theoretically in rock salt lead and tin chalcogenides (Delaire *et al.*, 2011 ▸; Li *et al.*, 2014 ▸; Lee *et al.*, 2014 ▸; Shulumba *et al.*, 2017 ▸). However, strong anharmonicity is not a necessary criterion for obtaining low thermal conductivity, and it is also important to acknowledge that the degree of covalency in a system will be important in determining its lattice thermal conductivity. It is clearly of interest to further probe relations between the extent of lone pair expression, thermal conductivity, structural disorder and anharmonic thermal motion.

## 4. Conclusions   

We have shown that MnSb_2_O_4_ has all the characteristics of a material with Sb(III) stereochemically active lone pairs. We observe significant contribution of antimony 5*s* and 5*p* states close to the Fermi level, and from the valence density, this is attributed to an antibonding configuration expressed as a stereochemically active lone pair. From real space descriptors such as the ELF and the Laplacian of the electron density, we found the positions of the antimony lone pairs, which were shown to avoid each other by inducing an angle between the lone pair and the interatomic line. This deflection was shown to increase with decreasing distance between antimony atoms by simulating the effect of pressure on the material. The chemical bonding in the material was shown to have a significant degree of covalency, which is important for our understanding of the lone pair formation, as the current theory requires a significant degree of orbital overlap. Analysis of the valence electron density suggested that the oxygen atom bonded to two Sb atoms was more involved in lone pair formation than the one bonded to only one Sb atom. This inspired a database search for structures with isolated and connected Sb*X*_3_ units (where *X* is a chalcogen), and showed that larger bond angles are generally found for isolated units. These observations suggest a degree of cooperative effect in the lone pair expression. Since heat conduction is normally largest along covalent bond directions, a stronger lone pair expression should lead to higher thermal conductivity, as is indeed the case for Sb*X*_3_ structures. Thus, it appears that for these chalcogenides, lone pair expression and thermal conductivity may be mainly related through the degree of covalency of the system, which affects the phonon dispersion, and not necessarily through strong anharmonicity, which decreases the thermal conductivity via phonon–phonon scattering.

## Supplementary Material

Supporting tables. DOI: 10.1107/S2052252520003619/lt5027sup1.pdf

## Figures and Tables

**Table 1:** Bond critical points and properties evaluated at these points *R* is distance between the atoms; *d*1 and *d*2 are the distances from the BCP to the first and second atom, respectively; ρ(*r*_b_) and ∇^2^ρ(*r*_b_) are the electron density and Laplacian of the electron density; *G*(*r*_b_), *V*(*r*_b_) and *H*(*r*_b_) are the kinetic, potential and total energy densities at the BCP; and ∊ is the ellipticity. The Sb—O2 bond marked by an asterisk is not shown in the structural figures, as it is significantly longer than the typical bond length.

	*R* (Å)	*d*1 (Å)	*d*2 (Å)	ρ(*r*_b_) (eÅ^−3^)	∇^2^ρ(*r*_b_) (eÅ^−5^)	*G*(*r*_b_) (Hartree Å^−3^)	*V*(*r*_b_) (Hartree Å^−3^)	*H*(*r*_b_) (Hartree Å^−3^)	*G*(*r*_b_)/ρ(*r*_b_) (Hartree e^−1^)	∊
Mn—O2	2.16	1.07	1.09	0.39	6.13	0.50	−0.57	−0.07	1.27	0.03
M—O1	2.18	1.08	1.10	0.35	5.69	0.45	−0.50	−0.05	1.26	0.04
Sb—O2	1.97	1.01	0.96	0.80	10.28	0.98	−1.24	−0.26	1.23	0.01
Sb—O1	2.02	1.03	0.99	0.70	9.01	0.83	−1.02	−0.20	1.19	0.05
Sb—O2*	2.84	1.49	1.35	0.14	1.33	0.09	−0.09	0.00	0.67	0.01
Sb—Sb	4.22	2.11	2.11	0.04	0.27	0.02	−0.01	0.00	0.38	0.08

**Table 2:** Integrated atomic properties Atomic charges (*Q*) are in units of electrons, volumes (*V*) in Å^3^ and integrated Lagrangian (*L*) in atomic units. Only the unique atoms from the structural point of view are shown. The only numerically significant differences between magnetically different atoms are the spin density related properties.

	*Q*	*V*	*L*
Mn	1.53	9.47	−9.1 × 10^−3^
Sb	1.89	23.0	2.1 × 10^−3^
O1	−1.30	14.3	1.5 × 10^−4^
O2	−1.35	14.3	3.3 × 10^−4^
