# Supplementary material for: Expression and interactions of stereochemically active lone pairs and their relation to structural distortions and thermal conductivity
Source: IUCrJ. 2020 Mar 31;7(Pt 3):480–9. doi: 10.1107/S2052252520003619 (PMC7201275; doi:10.1107/S2052252520003619)
Supplement: Supplementary file 1 [file m-07-00480-sup1.pdf]

IUCrJ

Volume 7 (2020)

Supporting information for article:

Expression and interactions of stereochemically active lone pairs and their relation to structural distortions and thermal conductivity

Kasper Tolborg, Carlo Gatti and Bo B. Iversen

S1. Geometry optimization

The full geometry, i.e. unit cell and fractional coordinates, were optimized starting from the structure from Roelsgaard *et al.* (2016) according to the information given in the manuscript. In Table S1, a comparison of the optimized structure with the 100 K experimental structure is given.

Table S1 Optimized geometry and experimental geometry. The numbers in parentheses are the percentage deviation from experimental values

Parameter	Optimized	Experimental
a (Å)	8.65577671 (-0.40 %)	8.6905
c (Å)	6.01823632 (+0.26 %)	6.0024
Mn	(0,0.5,0.25)	(0,0.5,0.25)
Sb	(0.17886,0.16577,0.5)	(0.17912,0.16801,0.5)
O1	(0.17847,0.32153,0.25)	(0.1795,0.3205,0.25)
O2	(0.40618,0.15253,0.5)	(0.4005,0.1450,0.5)
Mn-O1 (Å)	2.1847 (-0.83 %)	2.203
Mn-O2 (Å)	2.1602 (+0.85 %)	2.142
Sb-O1 (Å)	2.0203 (+0.94 %)	2.002
Sb-O2 (Å)	1.9710 (+1.91 %)	1.934

S2. ICSD search

Table S2 All structures with space group number and ICSD code used in the final analysis of bond angles in SbX_3 (X=O,S,Se) units. There are 77 structures in total with 137 unique connected SbX_3 units and 37 unique isolated SbX_3 units.

Chemical Formula	Space Group number	ICSD code	Connected units	Isolated units
$MgSb_2O_4$	135	4122	1	0
$Ba_3(SbO_3)_2$	2	413764	0	3
$Sr_5Sb_{22}O_{38}$	14	425765	5	0
$Na_3(SbO_3)$	217	23346	0	1
$LiSbO_2$	14	262075	2	0
$K_3(SbO_3)$	198	279579	0	1
$Cs_3(SbO_3)$	198	279580	0	1
KSb_3O_5	14	28493	2	0
$NaSb_5O_8$	2	28494	3	0
$Cs_2SbO_{2.5}$	8	411212	2	0
$Rb_2(Sb_8O_{13})$	11	412329	7	0
$Fe(Sb_2O_4)$	14	155152	2	0
$(Sb_4O_5)(ReO_4)_2$	2	165619	4	0
$Cu(Sb_2O_4)$	106	190731	1	0
Sb_2VO_5	62	203130	1	0
$MnSb_2O_4$	135	243444	1	0
$CoSb_2O_4$	135	262627	1	0
Sb_2VO_5	15	27800	1	0
$ZnSb_2O_4$	135	31996	1	0

$FeO(Sb_2O_3)$	135	4459	1	0
$Sb_2(MoO_6)$	2	59292	8	0
Sb_2WO_6	1	75595	2	0
$NiSb_2O_4$	135	86492	1	0
$Ba_4Sb_4Se_{11}$	58	31500	3	1
$BaSb_2Se_4$	14	32040	2	0
$Ba_3Sb_2Se_7$	15	429279	0	2
$KSbSe_2$	2	100125	1	0
$CsSbSe_2$	14	20773	2	0
$K_2(Sb_4Se_8)$	2	402886	1	0
$Rb_2(Sb_4Se_8)$	2	402887	1	0
Na_3SbSe_3	198	425125	0	1
$CsSb_2Se_4$	2	61220	1	0
$RbSb_3Se_5$	14	64672	3	0
$KSbSe_2$	1	660008	2	0
K_3SbSe_3	198	89607	0	1
Rb_3SbSe_3	198	89608	0	1
Cs_3SbSe_3	198	89609	0	1
$CuSbSe_2$	62	238476	1	0
Cu_3SbSe_3	62	401095	0	1
Ag_5SbSe_4	36	427307	0	1
Ag_3SbSe_3	62	427308	0	3
$SbCrSe_3$	62	84866	1	0
$Ca_2Sb_2S_5$	14	201044	1	1
$Sr_3Sb_4S_9$	33	201400	3	0
$Ba_8(Sb_6S_{17})$	13	26434	2	3
$BaSb_2S_4$	14	38372	2	0
$Ba_3Sb_2S_7$	15	429278	0	2
$Sr_6Sb_6S_{17}$	19	90094	6	0
$RbSbS_2$	1	200263	2	0
$CsSbS_2$	14	200798	1	0
$Cs_4Sb_{14}S_{23}$	2	20866	14	0
$Cs_2Sb_4S_7$	14	2193	3	0
$Rb_2Sb_4S_7$	2	2194	2	0
$K_2Sb_4S_7$	15	25329	1	0
$K(Sb_5S_8)$	7	410178	7	0
Li_3SbS_3	33	424834	0	1
Na_3SbS_3	198	425458	0	1
Cs_3SbS_3	198	426551	0	1
K_3SbS_3	198	426552	0	1
Rb_3SbS_3	198	426554	0	1
$Li_3S_{18}Sb_{11}$	2	433665	10	0
$RbSbS_2$	2	56788	1	0
$Cs_2(S_2)(Sb_4S_6)$	2	67976	1	0
$AgSbS_2$	9	16578	2	0
$CuSbS_2$	62	171051	1	0

$HgSb_4S_8$	15	174377	4	0
Ag_3SbS_3	161	181518	0	1
Ag_5SbS_4	36	290662	0	1
$Ag_3(SbS_3)$	14	33714	0	1
$Cu_3(SbS_3)$	19	403113	0	1
$MnSb_2S_4$	12	411178	2	0
$MnSb_2S_4$	62	56379	2	0
$CrSbS_3$	62	74601	1	0
Cu_3SbS_3	14	74901	0	2
$AgSbS_2$	5	85130	2	0
$FeSb_2S_4$	62	93911	2	0
$AgSbS_2$	15	94647	1	0
